# A Peculiar Case of Syphilitic Infection

**Published:** 1896-01

**Authors:** F. G. Moehlau

**Affiliations:** Clinical instructor in genito-urinary diseases; instructor in physiology, University of Buffalo


### Clinical Report.

### A PECULIAR CASE OF SYPHILITIC INFECTION.

By F. G. MOEHLAU, M. D.,

Clinical instructor in genito-urinary diseases; instructor in physiology, University of Buffalo.

IN THE latter part of June, 1895, an Italian applied to the university dispensary for treatment. Occupation, candy-maker; selling his goods on the corner of Broadway and Lafayette square at the time; age, 25; single. Examination showed a sclerotic chancre of three or four weeks' standing, patches in the mouth and throat, papular eruption on his back and inguinal lymphatics enlarged. He was treated for about a week, after which he never returned.

In the beginning of September, following, I was called upon to treat canker sore mouths in two girls, eight and twelve years respectively, and found, besides other signs, a sore resembling a chancre, in the younger girl, on the upper lip; in the older, on the fauces. After about four weeks I was informed that both children had a red rash on their backs and abdomens, which proved to be syphilitic and loss of hair followed. One cycle of inunction soon caused the disappearance of the general symptoms. At that time I accidentally thought of my patient, the candy-maker. The children were often in that neighborhood and I thought of a possible infection, which it truly proved to be, as the parents were both healthy and the children had bought candy of the Italian vender several times. About two or three weeks after having partaken of the sweets the sores developed. Since then, the children left the city with their parents and I have been unable to find out more about them. After speaking to the Italian once upon the subject of his disease I have not seen him since.

A similar case came under my observation, where a baker gave syphilis to his apprentice. The sore of the boy was on the anus. How the infection took place was not to be found out, as he claimed he only wore a pair of his partner's trousers.

